# Diagnosis and Management of Malnutrition in Patients with Heart Failure

**DOI:** 10.3390/jcm12093320

**Published:** 2023-05-06

**Authors:** Alberto Esteban-Fernández, Rocío Villar-Taibo, Mirian Alejo, David Arroyo, Juan Luis Bonilla Palomas, Montserrat Cachero, Clara Joaquin, Manuel Méndez Bailón, José Ángel Pérez-Rivera, Juan Carlos Romero-Vigara, Gema Somoza

**Affiliations:** 1 Cardiology Department, Severo Ochoa University Hospital, Calle Orellana s/n, 28911 Madrid, Spain; 2 Faculty of Health Sciences, Valencian International University, 46002 Valencia, Spain; 3 Endocrinology Department, Santiago University Clinical Hospital, 15706 Santiago de Compostela, Spain; rotaibo22@gmail.com; 4 Endocrinology Department, Hospital El Bierzo, 24404 Ponferrada, Spain; mirian_alejo@hotmail.com; 5 Nephrology Department, Gregorio Marañón General University Hospital, 28007 Madrid, Spain; dvdrry@gmail.com; 6 Cardiology Department, San Juan de la Cruz Hospital, 23400 Úbeda, Spain; jnlsbnll@hotmail.com; 7 Endocrinology Department, Germans Trias i Pujol Hospital, 08916 Badalona, Spain; mtdcachero@gmail.com (M.C.); clara.joaquim@gmail.com (C.J.); 8 Internal Medicine Department, San Carlos Clinical University Hospital, 28034 Madrid, Spain; manuelmenba@hotmail.com; 9 Cardiology Department, Burgos University Hospital, 09006 Burgos, Spain; jangel.perezrivera@gmail.com; 10 Faculty of Health Sciences, Isabel I University, 09003 Burgos, Spain; 11 Corella Health Center, 31591 Navarra, Spain; jcromerovigara@hotmail.es; 12 Geriatric Department, Gregorio Marañón University Hospital, 28007 Madrid, Spain; somoza.ge@gmail.com

**Keywords:** heart failure, malnutrition, cardiovascular, elderly

## Abstract

Heart failure is a disease with an increasingly greater prevalence due to the aging population, the development of new drugs, and the organization of healthcare processes. Malnutrition has been identified as a poor prognostic factor in these patients, very often linked to frailty or to other comorbidities, meaning that early diagnosis and treatment are essential. This paper reviews some important aspects of the pathophysiology, detection, and management of malnutrition in patients with heart failure.

## 1. Introduction

The prevalence of heart failure (HF) has continued to increase in recent years due to the aging population and the improvement in treatments and in healthcare organizations [1]. However, the high prevalence of cardiovascular (CV) risk factors in the developed world, closely related to a sedentary lifestyle and new nutritional and dietary patterns, also contributes to the increasing incidence of CV disease and the development of HF [2,3]. 

The prevalence of malnutrition in the European population varies according to the series, and increases with age, ranging from 10% to 29% [4,5,6]. In patients with HF, the incidence varies between 8% and 57%, depending on the level of severity [7]. The presence of malnutrition increases the mortality of these patients between 2- and 10-fold [8]; this may increase if frailty is associated, which occurs in 5–15% of cases [1,9]. Moreover, the development of HF has been directly related to the presence of obesity, whereby each 1 kg/m^2^ increase in the body mass index (BMI) entails an increased risk of HF of between 5% and 7% [10]. 

All patients with HF must be integrated into structured multidisciplinary care programs to reduce their morbidity and mortality [1,11]. In this comprehensive management, nutritional aspects must be addressed, the different malnutrition phenotypes identified, and nutritional treatment programs targeting prevention and reversal should be established, which could in turn impact prognosis. This paper will review different nutritional aspects of patients with HF, their nutritional requirements, the main tools for early diagnosis, and a number of strategies for the therapeutic approach. 

### 1.1. Definitions and Classification

In HF, nutritional disorders predominate by default, especially malnutrition, sarcopenia, and cachexia. Malnutrition is the state resulting from the lack of intake or malabsorption of nutrients that leads to altered body composition (decrease in fat-free mass) and in body cell mass, prompting a reduction in physical and mental function that leads to disease. Malnutrition can be the result of inanition, disease, or aging [12]. In the recently published guidelines on definitions and terminology in the Clinical Nutrition of the European Society of Clinical Nutrition and Metabolism (ESPEN), malnutrition is classified into three types [13]. Disease-related malnutrition (DRM) without inflammation is a form of malnutrition triggered by a disease in which inflammation is not among the etiologic mechanisms. These alternative mechanisms may include dysphagia, psychiatric conditions, such as anorexia nervosa, or malabsorption due to digestive disorders, such as protein-losing enteropathy, that can occur in HF. DRM with inflammation can be subclassified into DRM with chronic or acute inflammation depending on the degree of the inflammatory response induced by the disease. DRM with acute inflammation is typical of patients admitted to Intensive Care Units, such as coronary units, and of those who have undergone major surgery, such as cardiac surgery. In these cases, the combined action of elevated proinflammatory cytokine activity, increased catecholamine and cortisol production, insulin resistance, bed rest, and no or reduced food intake will lead to a decreased rapid depletion of energy and nutrient reserves that can quickly lead to malnutrition if adequate and early nutritional support is not provided. On the other hand, DRM with chronic inflammation usually appears in patients with advanced-stage chronic diseases such as chronic obstructive pulmonary disease (COPD), chronic kidney disease, or chronic HF. In these cases, systemic inflammation is usually mild, with serum C-reactive protein (CRP) concentrations rarely exceeding 40 mg/L. To define relevant inflammation in this scenario, a CRP of 5 mg/L is suggested as the lower limit. The importance of differentiating the type of DRE lies in the different pathophysiology and treatment of each one of them. Thus, while DRE without inflammation can usually be resolved with adequate nutrition, DRE with inflammation requires a multidisciplinary approach that includes intensive nutritional support, better control of the underlying disease, and rehabilitative treatment. 

Cachexia is defined as a multifactorial syndrome characterized by a severe loss of weight, muscle mass and fat mass, and by an increased protein catabolism as a consequence of an underlying disease that cannot be reversed with nutritional treatment alone [12]. Anker et al., in a study carried out in 1997, observed that a weight loss >7.5% in the absence of oedema, which they defined as cardiac cachexia, was an independent predictor of mortality. In this study, patients with cardiac cachexia had a mortality rate of 50% at 18 months, compared to 17% of patients without cachexia [14]. Since then, and up to the present, this definition of cardiac cachexia continues to be maintained as described in the latest ESPEN terminology guidelines [12]. However, in these guidelines, cachexia is considered to be synonymous with Disease-Related Malnutrition with chronic inflammation. Cardiac cachexia could be considered the tip of the iceberg of all the nutritional alterations that accompany chronic HF since, in these patients, we can also observe DRM without inflammation or DRM with acute inflammation during exacerbations, as well as multiple micronutrient deficiencies.

Most clinical studies in patients with HF do not adequately discriminate between the different states of malnutrition, since they are phenotypically very similar and often coexist. This would explain the differences in the effectiveness of interventions aimed at improving the malnutrition/cachexia complexes that have been observed in this pathology.

Sarcopenia is a progressive and generalized skeletal muscle disorder involving a decrease in muscle strength secondary to a reduction in muscle quantity and quality, often associated with poor physical performance. This muscle dysfunction can be detected both quantitatively (loss of muscle mass) and qualitatively (decreased muscle strength or physical performance) [15]. It is precisely this functional alteration that would seem to be most related to the severity of sarcopenia. The SICA-HF study estimated that the prevalence of sarcopenia in patients with HF and a reduced left ventricular ejection fraction (LVEF) was 20 times higher than in healthy adults of the same age [16].

Sarcopenic obesity is a clinical and functional condition defined by the coexistence of excess adiposity accompanied by a decrease in muscle mass and in related strength and functionality. The underlying mechanisms include low-grade chronic inflammatory activity and muscle inactivity secondary to the underlying condition. Its coexistence with HF is very common, particularly in HF with preserved LVEF. In these cases, there is an important limitation of exercise capacity [12,17].

### 1.2. Prognosis and Consequences of Malnutrition in HF

Malnutrition has an unfavorable prognostic effect on HF. A study including discharged patients after hospital admission due to acute HF found that a malnourished patient was four times more likely to die during follow-up than a patient with a normal nutritional status [18]. Similar findings were reported in another study in a population of 3386 patients with HF, evaluated on an outpatient basis which identified malnutrition as an important predictor of mortality at one year [19]. The role of malnutrition could be related to the metabolic alteration present in malnourished patients, characterized by an increase in inflammatory cytokines and a decrease in protein synthesis.

Sarcopenia is one of the main causes of exercise intolerance and worsening of respiratory and functional function in patients with HF. It causes a deterioration in the quality of life and is associated with a longer hospital stay, more frequent readmissions, and a poorer overall prognosis [20].

## 2. Causes and Pathophysiological Mechanisms of Malnutrition in HF

**Figure 1:**
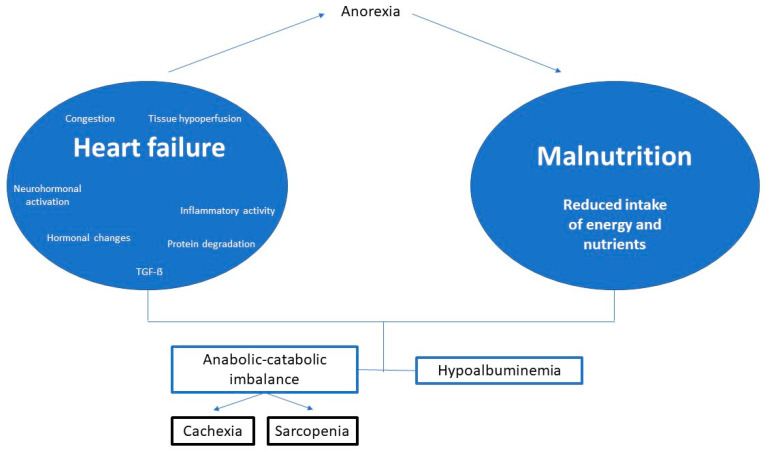
Summary of the pathophysiology of malnutrition in heart failure. Modified from De Cruz-Jentoft A.J., et al. [15].

In patients with HF, the hormonal dysregulation and the neurohormonal and inflammatory activation, typical of the disease, produce an anabolic-catabolic imbalance favoring catabolism. In addition, the HF itself will be a cause of malnutrition, by altering the appetite and food intake and even through nutrient malabsorption (Figure 1).

### 2.1. Hemodynamic Alterations

The loss of viable myocardium and contractile force triggers an increase in the central venous pressure and tissue hypoperfusion, particularly in the skeletal muscle, which could trigger nutritional and metabolic disorders [21] such as, (a) peripheral hypoperfusion, in conjunction with reduced physical activity, generate skeletal myopathy [22]; (b) intestinal congestion which can cause satiety and nausea (reducing intake) [23], and promote enteric albumin loss, producing hypoalbuminemia [24]; and (c) the increase in venous pressure favors the leakage of the albumin into the extravascular space, contributing to the development of hypoalbuminemia [25].

### 2.2. Neurohormonal and Adrenergic Activation and Inflammation

Neurohormonal, adrenergic, and inflammatory activation are involved in the progression of HF [26,27,28]. Sympathetic activation increases basal metabolism and resting energy expenditure [23,24]. An increase in adrenaline and noradrenaline has been detected in cachectic patients, demonstrating a direct association between noradrenaline and cachexia. Precisely beta-blocker therapy has been shown to promote weight gain, which is greater in cachectic patients [29]. The activation of the renin-angiotensin-aldosterone system (RAAS) is also greater in cachectic patients [30]. Studies with RAAS inhibitors have pointed to a reduction in the risk of suffering weight loss [31].

In terms of inflammation, TNF-α has a catabolic effect, producing anorexia, exercise intolerance, apoptosis, proteolysis, and increased plasma leptin concentration, which, in turn, reduces food intake and increases resting energy expenditure [32]. IL-6 increases the endothelial vasoactive peptides and can induce proteolysis, muscle atrophy, and weight loss [32]. In addition, it inhibits the hepatic synthesis of the albumin and, together with other inflammatory cytokines, increases vascular endothelium permeability, favoring the leakage of the albumin into the extravascular space (hypoalbuminemia) [33].

### 2.3. Hormonal Imbalance

The anabolic-catabolic imbalance in patients with cardiac cachexia has been related to an increase in cortisol (catabolic effect) and a decrease in dehydroepiandrosterone (anabolic effect) [32]. Ghrelin stimulates the appetite and has an anabolic effect, stimulating growth hormone (GH) [34]. Patients with cardiac cachexia appear to be resistant to both ghrelin and GH [35].

### 2.4. Protein Degradation

Various factors influence the loss of muscle mass and are linked to hormonal dysregulation, neurohormonal and inflammatory activation, and the increased activity of the protein degradation pathways [34].

### 2.5. Transforming Growth Factor Beta Family

Myostatin is a negative muscle growth regulator that plays an important role in the development of skeletal muscle mass. It has been seen to be over-expressed in patients with HF, which has been related to a loss of muscle mass [36]. Growth differentiation factor-15 (GDF-15), previously associated with anorexia, nausea, weight loss, and cachexia in cancer patients, is also elevated in patients with HF and has been associated with a lower exercise capacity, lower BMI, and higher mortality [37].

## 3. Nutritional Needs of Patients with HF

The evidence about the nutritional needs of patients with HF is scarce and the HF guidelines do not provide specific nutritional recommendations [1,38]. However, the quality of nutritional contribution in patients with HF is frequently deficient, which may be associated with adverse clinical outcomes. The evidence obtained in recent years supports the idea that adequate protein supplementation increases muscle synthesis, and this effect is enhanced when combined with physical exercise. Table 1 shows some of the main nutritional recommendations for patients with HF.

### 3.1. Caloric Requirements

Energy intake should be adjusted to the patient’s nutritional status. Patients with cardiac cachexia have a high energy expenditure, whereas HF patients without cachexia have a 10–20% decrease in total energy expenditure due to reduced activity [39]. An adequate caloric intake is known to improve the quality of life. However, an excess energy intake may cause HF decompensation owing to greater physiological stress [40].

An intake of 22 kcal/kg per actual weight is recommended in normonourished patients and 24 kcal/kg per actual weight with malnutrition, with the physical activity factor being subsequently multiplied to estimate the actual energy needs [41]. In general, feeding should begin with a low kcal/kg per actual weight ratio, nutrients should be provided slowly and progressively, and large volumes should be avoided [40].

### 3.2. Protein Requirements

Protein intake does not differ from that of healthy people and may be increased in the event of protein losses due to malabsorption or nephropathy. Renal functionality should be taken into account owing to the frequent association of cardiorenal syndrome (CRS). Adequate protein supplementation increases muscle synthesis, an effect that is enhanced if combined with physical exercise. Protein intake should be individualized, with at least 1.1 g of protein/kg per actual weight prescribed to prevent catabolism [41], although some groups suggest a somewhat higher contribution, from 1 to 1.5 g/kg per weight, with high biological-value proteins to promote muscle mass recovery [39]. Other investigations relate a protein intake of 1.1–1.4 g/kg/day to a positive nitrogen balance, while a protein intake of between 1.0 and 1.1 g/kg/day was associated with a negative nitrogen balance (normonourished and malnourished patients) [41].

### 3.3. Carbohydrates (CHO) and Fats

There are no specific recommendations, except in cases in which parenteral nutrition is required (CHO intake <4–5 mg/kg of body weight /min) [40] or in the context of HF and obesity, when a hypocaloric diet may be considered in order to achieve a weight loss of 5–10% with a lower proportion of CHO (CHO 40%/proteins 30%/fats 30%) [2]. The recommendations of these macronutrients in the general population could be applied to most patients with HF and range between 45% and 65% of CHO intake, and 20% and 35% of total fats (<10% saturated and polyunsaturated fatty acids) [42].

### 3.4. Fluids and Sodium

The recommendations in patients with HF have traditionally focused on restricting fluids and sodium (Na) in the diet; however, there are no solid data that demonstrate better outcomes with these measures. In patients with severe HF, a maximum fluid intake of 1.5–2 L/day is recommended, particularly in the presence of hyponatremia, to reduce congestive symptoms [1,2,38,41].

Regarding the consumption of Na, an intake of 2–3 g/day is recommended for most patients [38,41], and a salt intake greater than 5 g/day should be avoided (2 g of Na) [1]. The SALT-HF trial in patients with stable HF showed that the strict reduction of Na below 1.6 g/day did not reduce CV events compared to a low-Na diet (2 g/day) [43].

### 3.5. Other Micronutrients

A restrictive nutritional recommendation can lead to an insufficient intake and a potential deficiency of certain micronutrients, such as calcium, magnesium (Mg), zinc, iron (Fe), thiamine, vitamins D, E and K, folic acid, and selenium, which may be increased due to renal losses secondary to the use of diuretics. Severe deficiencies of certain micronutrients can cause functional cardiac alterations, sometimes serious [44,45]; hence, they must be supplemented (through nutritional intake or oral supplements) in the case of deficiency [39,46,47].

An insufficient intake, and the continued use of diuretics, may cause fat-soluble vitamin (A, D, E, K) deficiency. Vitamin D deficiency tends to be common, although supplementation has not shown an improvement in quality of life, biochemical parameters, CV death, or hospitalization [1,38,41]. In some cases, calcium supplementation, alone or routinely combined with vitamin D, has been associated with decreased survival and an increased CV risk in older patients with mild-to-moderate aortic stenosis [48]. Therefore, the general recommendation would be to supplement only in cases of deficiency.

Water-soluble vitamin deficiency (B1-B6-B12) is rare. A B1 (thiamine) deficiency may be observed in relation to alcohol abuse or to the use of high-dose diuretics, possibly on account of their water solubility and low renal reabsorption [2]. A relationship between thiamine and cardiac function has also been observed, and there may be a positive association between thiamine replacement and the improvement of LVEF in patients with HF [49].

The European HF guidelines recommend evaluating the use of omega-3 polyunsaturated fatty acids (PUFA) in patients with HF (level of evidence B) [1,38]. The supplementation with 1–2 g/day could reduce the risk of hospitalization or CV death [34,50,51], although the evidence is controversial [52,53]. The American guidelines establish that it is reasonable to use them as a complementary treatment to reduce mortality and hospitalizations unless contraindicated [38].

Coenzyme Q10 concentrations are decreased at a myocardial level in these patients, which could be related to higher mortality. Doses of 60–300 mg/day have been studied, and suggest an improvement in functional class, LVEF, exercise capacity, and survival, although the evidence is controversial [54,55,56].

Iron (Fe) deficiency is frequent in patients with HF, regardless of the existence of anemia, which has been associated with a poorer prognosis. The intravenous intake of Fe with ferric carboxymaltose to correct the Fe deficiency is indicated in patients with HF and LVEF < 50% to reduce hospitalizations for HF and to improve functional class [1,2], although no benefit has been demonstrated with oral supplementation.

Finally, other nutrients, such as L-carnitine, creatine, and taurine, may be effective, particularly if combined with each other [40].

## 4. Nutritional Particularities in Some Subpopulations of Patients with HF

### 4.1. Older Patients

Older people present a series of peculiarities, such as the coexistence of comorbidities, aging-related physiological changes, the frequently atypical presentation of several pathologies (such as *delirium* as the initial manifestation of an acute coronary syndrome), and the high prevalence of geriatric syndromes (frailty, falls, polypharmacy, cognitive impairment, etc.). These characteristics render a comprehensive geriatric assessment necessary.

The risk of malnutrition increases with age, frailty, and comorbidity. So much so that its prevalence is around 40% in the community environment and can exceed 70% in the context of acute HF decompensation [57,58,59]. Its presence is also associated with a higher risk of mortality, disability, and institutionalization [60]. All elderly people with HF should be screened periodically for malnutrition, irrespective of their care environment [60], in order to establish a care plan tailored to each patient’s individual needs.

### 4.2. Diabetes Mellitus

The association between HF and diabetes (DM) is frequent, and from the nutritional standpoint, there are two clinical profiles of patients, overweight or obese patients and elderly patients with malnutrition.

The recommended diet for diabetic patients with HF is the DASH (*Dietary Approach to Stop Hypertension*) or the Mediterranean diet. In the case of overweight or obese type-2 diabetics with HF, weight reduction will be the therapeutic objective. Caloric restriction and weight loss will produce an improvement in carbohydrate metabolism, a reduction in insulin resistance, and an improvement in pancreatic beta cell function. All these measures can achieve a reduction in the dose of insulin and further weight loss. The use of sodium-glucose cotransporter type-2 inhibitors (SGLT2) has shown a prognostic benefit regardless of the existence of DM, also accompanied by weight loss [1]. Other drugs, such as glucagon-like peptide-1 receptor agonists (GLP-1-RAs), have shown significant weight loss and a reduction in CV events in diabetics with obesity [61].

On the other hand, older patients with HF, DM, frailty, and malnutrition may be at a greater risk of hypoglycemia. Drugs that can cause hypoglycemia should be avoided, and oral nutritional support should be based on diets with an adequate protein content, heart-healthy fats, and trace elements with a low proportion of carbohydrates.

### 4.3. Cardiorenal Syndrome (CRS)

Chronic kidney disease (CKD) can coexist with HF in 25–40% of patients [62]. CRS complicates nutritional management since patients with nephropathy have circumstances that contribute to malnutrition, uremic anorexia, inflammation, and hormonal and electrolyte disturbances, which promote a hypercatabolic state.

The International Society of Renal Nutrition and Metabolism has established the diagnostic criteria of the “protein-energy wasting” syndrome [63]. Nonetheless, a combination of different diagnostic tools is recommended, such as dietary surveys, anthropometric and biochemical parameters, and other specific tests.

Protein intake is particularly relevant in nutritional management. In the advanced phases of CKD, moderate protein restriction is recommended, whereas a marked increase in protein intake is required in renal replacement therapy. Similarly, clearly-defined specifications that moderate the intake of potassium (K) and phosphorus will be necessary to avoid short- and long-term complications. However, recent studies, such as the NDD-CKD, question the relevance of strict K diet restrictions in patients with CKD or on dialysis [64].

### 4.4. Anticoagulated Patients

Malnutrition and low body weight are risk factors for bleeding in anticoagulated patients. In a recent study that included patients with atrial fibrillation (AF) treated with vitamin K antagonists (VKAs), malnutrition was associated with a 3-fold increased risk of bleeding [65]. Other authors have found an association between hypoalbuminemia and the risk of bleeding due to an increase in the warfarin-free fraction in serum [66]. A restriction on foods rich in vitamin K has usually been recommended in these patients (green leafy vegetables or some fruits), although the evidence shows that it is more important to develop healthy dietary habits and to maintain a stable intake of these foods [67]. Low body weight, as well as CKD and age, are factors limiting the dosage of direct-acting anticoagulants and must, therefore, be factored in when these drugs are prescribed [68].

## 5. Diagnosis of Malnutrition

The diagnosis of malnutrition in patients with HF is a veritable challenge and usually requires the combination of several tools.

### 5.1. Nutritional Screening

Different nutritional screening tools have been used, such as the Mini Nutritional Assessment (MNA) and the MNA-short form (MNA-SF), the Malnutrition Screening Tool (MST), the Malnutrition Universal Screening Tool (MUST), and the Short Nutritional Assessment Questionnaire (SNAQ). Although none of them are regarded in the reference test, [57] one of the most widely used ones is the MNA-SF, which has shown better sensitivity and specificity in screening for malnutrition than the MUST and MST in these patients [69].

### 5.2. Diagnosis of Malnutrition

There is no consensus on the best method to assess the nutritional status of patients with HF. Several studies have shown that nutritional assessment methods, based almost exclusively on biochemical and immunological markers, are not the most appropriate [46]. For this reason, structured tools that allow a global assessment of nutritional statuses, such as the VGS or the MNA, should be used, as well as a combination of analytical and anthropometric parameters (Table 2).

A recent study showed that the MNA was a better prognostic marker of mortality than the VGS, and it also correlated with quality of life and physical function [46]. Other studies have also shown a relationship between malnutrition detected by the MNA and mortality, which is why it is recommended for the nutritional evaluation in these patients [57]. The *Global Leadership Initiative on Malnutrition* (GLIM) criteria were published with a view to standardizing the diagnosis of malnutrition in adults. Although the evidence for using these criteria in HF is still limited, their use may be extended in the future [70,71]. However, in a comparative study between the MNA and GLIM criteria, it transpired that the MNA is a better prognostic predictor of mortality and readmissions than the GLIM criteria [72].

The different components of nutritional assessment and body composition techniques are detailed below (Table 3).

#### 5.2.1. Biochemical Markers

Serum visceral proteins, such as albumin (half-life 14–21 days) and prealbumin (half-life 2–3 days), have been used as markers of the nutritional status in patients with HF, in addition to their having been identified as prognostic markers. Notwithstanding, they do have limitations, as they can be altered by systemic inflammation or congestion. Albumin behaves as a negative acute-phase reactant since its synthesis is suppressed in inflammatory states. Therefore, it should always be considered in combination with the evaluation of C-reactive protein (CRP). Prealbumin is less sensitive to hydration, and for this reason, it is postulated as a better marker of protein malnutrition. It is also a negative acute-phase reactant and should, therefore, be considered in conjunction with CRP. However, unlike albumin, and in view of its short half-life, it can be used to monitor short-term nutritional status changes [2].

#### 5.2.2. Anthropometry

These are the most commonly used methods as they are the most economical and accessible. They include weight, height, BMI, folds, and perimeters. In HF, subclinical volume overload complicates the assessment of weight loss and can lead to a falsely-elevated BMI. Moreover, rapid weight changes are often caused by changes in fluid volume due to the worsening of the HF or in response to therapy. Skin folds and arm muscle circumference (AMC) tend to be more accurate for determining nutritional status and body composition since they are more independent of the weight fluctuations caused by edema [46]. The measurement of skin folds using calipers evaluates the subcutaneous fat and makes it possible to estimate the percentage of total body fat through differential equations. The most widely used equation is the *Durnin-Womersley*, which requires the measurement of the four folds: bicipital, tricipital, subscapular, and suprailiac. As a complement to the measurement of skin folds, the AMC and arm muscle area measurements are used to assess fat-free body mass.

Waist circumference is directly correlated with the intra-abdominal fat content and, in particular, with visceral fat. It makes it possible to establish cut-off points that can be used to evaluate and predict obesity-related complications such as changes in the CHO metabolism, CV disease, and risk of mortality, even in patients with normal weight [61].

#### 5.2.3. Body Composition

The body composition (BC) assessment has proven its usefulness in determining the nutritional status and for planning the most appropriate nutritional support, making it a key tool in nutritional diagnosis. There are numerous methods, the most outstanding of which are bioelectrical impedance (BIA), dual-energy X-ray absorptiometry (DXA), computed tomography (CT), magnetic resonance imaging (MRI), and muscle ultrasound.

BIA is a fast, safe, economical, and easy-to-use option that provides an estimate of body composition based on mathematical calculations. However, it is contraindicated in patients with cardiac devices and is of limited use in patients with abnormal blood volume, such as HF [73]. This problem can be corrected by using a bioelectrical impedance vector analysis (BIVA).

DXA is an easy method to use, with minimal radiation exposure and a lower cost than CT or MRI. It can be used to assess muscle, adipose, and bone compartments; however, major changes in the hydration status (>5%) can overestimate fat-free mass, making it an unreliable tool for evaluating the muscle compartment in patients with HF [74].

CT and MRI may be considered alternatives for calculating the fat mass and fat-free mass; however, they are expensive and require highly-trained personnel. Muscle ultrasound is advocated as a measurement of muscle mass due to its simplicity, low cost, availability, and good correlation with the data obtained by magnetic resonance. Some studies have even shown its potential usefulness for the diagnosis of sarcopenia [75]. Currently, its main limitation is the lack of universally defined cut-off points [46].

### 5.3. Screening and Evaluation of Sarcopenia

**Figure 2:**
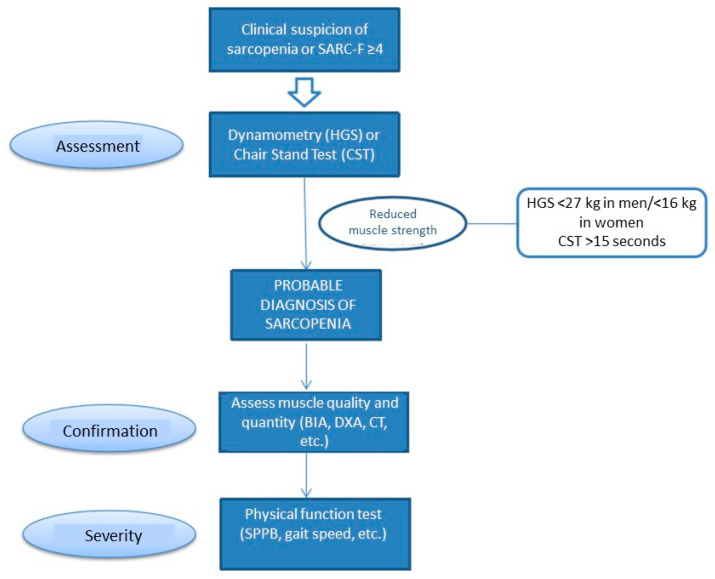
Screening and assessment of sarcopenia. Modified from De Cruz-Jentoft A.J., et al. [15].

Screening for sarcopenia is recommended when the patient presents a risk of malnutrition or symptoms consistent with sarcopenia (falls, weakness, slow walking speed, difficulty getting up from a chair, or loss of muscle mass) [15]. The SARC-F test is recommended for screening. This questionnaire can be self-administered and consists of five questions based on the patient’s perception of their limitations in terms of strength, ability to walk, get up from a chair, climb stairs, or falls (Figure 2).

Muscle strength can be assessed using a handgrip strength measured by dynamometry. This has been associated with a higher rate of postoperative complications and increased mortality in patients with advanced HF [76]. Muscle quantity and quality should be assessed using a BIA or imaging techniques, and physical performance by means of functional tests such as the *Short Physical Performance Battery* (SPPB).

## 6. Prevention and Nutritional Treatment

There are no specific nutritional recommendations for patients with HF, and an adequate and individualized eating plan must be established, accompanied by physical exercise and taking comorbidities and disease evolution into account [1,12]. However, certain healthy dietary patterns are recommended to promote CV health (Table S1 of the Supplementary Material). These patients may also present aspects that may interfere with their ability to consume an adequate diet, thereby requiring some specific recommendations (Table 4) [1,41,42].

Some studies suggest a protective effect of the Mediterranean diet or the DASH diet, with a reduction of up to 50% in the risk of developing HF in healthy subjects [77,78,79,80,81,82]. The DASH diet is characterized by promoting foods rich in K and limiting Na and total fats. Its main effect at the cardiac level is the lowering of blood pressure, which reduces the risk of developing HF. In addition, the abundant supply of antioxidants could improve cardiac contractility [80]. In the GOURMET-HF trial, conducted on older adults discharged from the hospital with HF, patients with a better adherence to the DASH pattern presented fewer symptoms, hospitalizations, and improved functional capacity [82].

The Mediterranean diet also promotes a higher intake of unsaturated fatty acids (fish, olive oil, and nuts). The MEDIT-AHF study, carried out in patients after an acute episode of HF, showed that a greater adherence to this diet reduced the rate of readmissions and does not influence long-term mortality [79]. Other studies have shown a decrease in HF incidences in subjects with a greater adherence to the Mediterranean pattern [83,84]. Although both dietary patterns share certain aspects, each one has its own particularities (Table S2 of the Supplementary Material).

Patients with HF who are overweight or who have grade 1–2 obesity seem to have a better prognosis than those with greater obesity or normal weight, and low-weight patients have a poorer prognosis. This paradox of obesity could be explained by the nutritional and inflammatory state, the different body composition, and the evolutionary stage of the disease [85]. However, obesity is a risk factor in the development of HF and it would appear that lean mass, rather than fat mass, has a protective effect. Therefore, a normocaloric diet should be established in patients with a healthy general dietary pattern and without malnutrition, and long-term caloric restriction should only be implemented in patients with significant obesity.

### Artificial Nutritional Support

Artificial nutritional support (ANS) is an essential therapy in the management of malnutrition when patients do not meet their requirements with natural food or when it is not safe and can improve the nutritional status in patients with HF. ANS is indicated in patients who are expected to have an insufficient oral intake for a period of more than 5–7 days, but in patients with malnutrition or an acute catabolic situation, this period is shortened and earlier initiation is advised.

There are several modalities of ANS, including oral, tube feeding (enteral), and parenteral nutrition. Oral nutritional supplementation (ONS) will be the first choice in patients who require ANS, unless it is contraindicated, and will usually be carried out with products designed to supplement ordinary food.

When is not possible to meet requirements with a natural oral diet and ONS, or when the oral route is not safe, the most appropriate nutritional support modality will be enteral tube nutrition, as it is more physiological, maintains the integrity of the intestinal mucosa, and is associated with fewer or less severe complications. Indications for enteral tube feeding include situations where oral intake is impossible (coma, mechanical swallowing impairment), or not advisable (dysphagia, low level of consciousness) and also situations where oral intake is insufficient (anorexia, situations with increased requirements). On the other hand, enteral tube feeding has some absolute contraindications (incoercible vomiting, digestive hemorrhage, paralytic ileus, intestinal obstruction, digestive perforation) or relative contraindications (upper jejunal fistulae, acute inflammatory bowel disease, short bowel syndrome with remaining bowel less than 50 cm, severe acute pancreatitis). These contraindications may be permanent or temporary and should be re-evaluated [86]. Patients with advanced HF associated with cachexia, malnutrition, or a risk of malnutrition may require nutritional intervention with enteral feeding when ONS, associated with natural feeding, is not safe or sufficient [40]. It should be initiated in the first 24–48 h post-admission to the cardiology critical care unit, and it should be carried out prudently due to the risk of intestinal ischemia, the objective being to reach 25–30 kcal/kg/day in the first week [87,88]. Enteral nutrition is not indicated for the prophylaxis of cardiac cachexia.

In cases where enteral nutrition is contraindicated, parenteral nutrition (PN) is indicated. PN would be contraindicated in the terminally ill patient, in clinical situations where the risks outweigh the benefits, when nutritional support is expected for <7 days, and especially when there is a functioning bowel that would allow enteral nutrition to be instituted. In patients with heart failure, PN may be necessary in cases of hospitalization for cardiogenic shock or in the context of cardiac surgery, alterations in intestinal permeability and absorption, patient instability, and volume limitations (provided that the indication criteria are met) [40]. Early PN is recommended when the provision of enteral nutrition is not feasible or sufficient in malnourished subjects or in those with a high risk of malnutrition [87].

Since there is no specific evidence for this group of patients, the nutritional support recommendations for critically ill and perioperative patients are usually followed [2,87,88]. The recommendations for artificial nutrition in critical and coronary patients were published in 2011 and were similar to the previous ones [89].

ANS required a mixture of a varied and balanced quantity of macro and micronutrients sufficient to cover the patient’s nutritional needs. It can be presented as complete formulas, as caloric, protein or mixed supplements, isolated nutrients, or as a mixture of two or more nutrients commonly known as modules. The objective of ANS will be to meet macro and micronutrient requirements while considering that these patients may have other associated conditions that require modifications (such as DM or CKD). Despite the scant evidence available, and the fact that the results of the studies on the composition of these formulas are very heterogeneous, the selection of the nutritional formula must be individualized (Table 5).

There have been few clinical trials addressing nutritional interventions in patients with HF, although some have yielded relevant results. In the PICNIC [90] study, conducted on HF patients hospitalized with malnutrition, the patients were randomized to receive conventional treatment versus conventional treatment combined with an individualized nutritional intervention (diet optimization or the use of oral nutritional supplements). The individualized nutritional intervention was shown to reduce the risk of total mortality by 63% and readmissions for HF by 63%. In a systematic review [91], the impact of nutritional intervention in HF patients who were malnourished or at risk of malnutrition was evaluated, with an average weight gain of 3.8 kg being observed. The EFFORT [92] study, carried out in patients with nutritional risk hospitalized with HF, assessed the impact of individualized early nutritional intervention on frailty, functionality, and recovery from malnutrition, and a 66% reduction in all-cause mortality at 30 days was observed compared with conventional hospital feeding. These findings support screening and individualized nutritional support at hospital admission, although there are no specific recommendations for the treatment of malnutrition in patients with HF, particularly in the outpatient setting [91,93,94].

However, evidence is scarce regarding the efficiency of nutritional intervention in this type of patient. Based on the EFFORT study results, Schuezt et al. used a Markov model to estimate the cost-effectiveness of individualized nutritional support in hospitalized patients with chronic HF. With nutritional support, they modeled an additional 5.77 life days, being the overall incremental cost-effectiveness ratio for nutritional support versus no nutritional support 2625 Swiss Francs (SF) per life day gained. However, in terms of complications, patients receiving nutritional support had a cost savings of 6214 SF and an additional 4.11 life days without complications, with an incremental cost-effectiveness ratio for avoided complications of 1513 SF per life day gained. Thus, the economic analysis concluded that in-hospital nutritional support for chronic HF patients increased life expectancy at an acceptable incremental cost-effectiveness ratio [95].

## 7. Drug Treatment and Interactions

Polypharmacy is frequent in patients with HF and is related to malnutrition (Table 6).

Multiple pharmacological interactions, adverse effects related to polypharmacy (nausea, dysgeusia, anorexia), as well as the diminished absorption rate of micronutrients, are all factors that contribute to its appearance.

Several drugs, such as diuretics or RAAS inhibitors, have been associated with a reduction in zinc levels, giving rise to dysgeusia, anorexia, and an increased risk of malnutrition [3]. For this reason, zinc levels should be monitored frequently in patients with HF and at risk of malnutrition, and adequate supplementation should be provided when a deficiency is detected [96]. Loop diuretics have also been associated with a decrease in thiamine levels, particularly with frequent alcohol consumption [97]. Chronic treatment with loop and thiazide diuretics is also associated with a higher risk of hypokalemia, which may increase arrhythmic events and mortality [98]. In patients with hypokalemia, a diet rich in K (spinach, tomato, avocado, banana) and Mg (nuts, vegetables, pumpkin seeds), as well as the use of certain drugs and supplements, can help to correct K levels. In patients with hypomagnesemia, especially if it is mild, the arrhythmic risk is low, and higher in the case of hypermagnesemia. Oral salt supplementation can be used in hypomagnesemia, although special caution should be exercised in patients with advanced CKD [99].

Finally, the use of proton pump inhibitors (PPIs), as well as metformin, is associated with vitamin B12 deficiency and, in the case of PPIs, Fe, and Mg, malabsorption [100]. The prolonged use of statins has been associated with a significant decrease in coenzyme Q10, α-tocopherol, β-carotene, and lycopene, a deficiency of which contributes to the appearance of statin-induced myopathy [101].

**Table 6:** Summary of the main pharmacological and nutritional interactions.

Disorder	Drugs	Monitoring	Treatment
Zinc deficiency	Loop diuretics Thiazide diuretics ACEI/ARA-II	Not recommended as routine [102] Assess whether dysgeusia and treatment with ACEI/ARA-II [96,103]	No routine supplementation [102] Assess whether dysgeusia and treatment with ACEI/ARA-II [96,103]
Thiamine deficiency	Loop diuretics	Not recommended as routine [102] Assess whether alcoholic cardiomyopathy [103]	No routine supplementation [102] Assess for alcoholic cardiomyopathy [103]
Hypokalemia	Loop diuretics Thiazide diuretics	Every 3–6 months if levels are stable [103] Every 2–4 weeks if the dose start/change: Loop diuretics/thiazides, K supplements (until stability) [103]	Supplementation if there is a deficiency or tendency to hypoK or high doses of diuretic [103] Form of supplementing [103] Potassium-rich diet Pharmacological supplements Optimize ACEI/ARA-II/ARNI, ARM if indicated
Hyperkalemia	ACEI/ARA-II/ARNI ARM	Every 3–6 months if levels are stable [103,104] Every 2–4 weeks if the dose start/change: IECA/ARA-II/ARNI, ARM, potassium supplements (until stable) [9]	Low potassium diet [103] Potassium binders [103,105] Caution with ACEI/ARA-II/ARNI/ARM if kidney failure or K > 5 mEq/L [102]
Hypomagnesemia	Loop diuretics	Every 3–6 months if levels are stable [99,103] Every week if the dose start/change (until stability) [99,103]	Supplementation if there is a deficiency or tendency to hypoMg and high doses of diuretic [99,103] Form of supplementing [99,103] Mg-rich diet Pharmacological supplements
Vitamin B12 deficiency	PPI Metformin	Recommended as routine if metformin [96,101]	Supplementation if deficiency [96]
Iron deficiency	PPI	Recommended in all patients with HF [103]	If there is iron deficiency (Ferritin <100 µg/L, or 100–300 µg/L + TSI <20%) with/without anemia, give intravenous iron (oral iron not effective) [103]
Coenzyme Q10 deficiency	Statins	Not recommended as routine	Supplementation is not recommended, although there are trials that suggest that it could improve mortality and hospitalizations due to HF [1]

HF: heart failure; ACEI: angiotensin-converting enzyme inhibitors; ARA-II: angiotensin II receptor antagonists; MRA: mineralocorticoid receptor antagonists; ARNI: angiotensin II and neprilysin inhibitors; PPIs: proton pump inhibitors; GFR: glomerular filtration rate; STI: transferrin saturation index; K: potassium; Mg: magnesium.

## 8. Physical and Functional Rehabilitation

Structured and personalized physical rehabilitation is an essential component for reversing sarcopenia in patients with advanced HF, and resistance training is particularly effective for patients with anaerobic limitations, leading to an improvement in muscle mass and strength, particularly in the lower body [2]. In patients with HF, a physical training program improves exercise tolerance and quality of life and reduces readmissions for HF, especially in patients with a reduced LVEF. However, the evidence on the reduction in all-cause mortality is controversial and the benefits have been observed particularly in programs with a follow-up of more than 12 months in patients with a reduced LVEF [102,103,104,105].

Cardiac rehabilitation has a class I indication with an evidence level A in all patients with HF; hence, all patients should be included in a rehabilitation program targeting patients with HF [1,38]. The main recommendations are listed in Table 7 [106,107,108].

## 9. Discussion

The detection and treatment of malnutrition in patients with HF is key, due to its high prevalence and its negative consequences on the prognosis of the disease, including deterioration in quality of life, increased hospitalizations, and increased mortality.

The alteration of the nutritional status that can appear in HF includes various clinical pictures such as DRM without inflammation, with acute inflammation or with chronic inflammation, cachexia, sarcopenia, or sarcopenic obesity. Establishing the correct diagnosis of the type of alteration present in the patient is essential to correctly guide the treatment, since in patients with inflammation, nutritional treatment will not be sufficient to improve the patient’s condition and will require a multidisciplinary approach that also includes rehabilitation treatment and good management of the underlying disease.

However, the diagnosis of malnutrition in HF is a challenge since there is little evidence on the ideal diagnostic method and anthropometry or classical biochemical markers are less appropriate in the context of volume overload associated with HF. In general, it would be more appropriate to use methods that allow a more global assessment of the nutritional status, such as MNA, GSA, or GLIM criteria, and to rely on other tools such as body composition methods (BIA, CT, or muscle ultrasound).

The nutritional approach to HF should promote dietary patterns with proven benefits in CV disease and also in HF, such as the Mediterranean diet or the DASH diet. Likewise, it is important to record in the clinical history the symptoms or drugs that may interfere with intake by producing anorexia, early satiety, chewing or swallowing difficulties, dyspepsia, micronutrient alterations, etc., in order to adapt nutritional recommendations and supplement deficits.

Artificial nutritional support will be indicated when the natural diet does not meet the patient’s requirements or when the patient cannot be safely fed orally. Nutritional intervention in hospitalized patients with HF and malnutrition has been shown to reduce mortality and readmissions significantly, so we should routinely incorporate nutritional screening of hospitalized patients with HF. Currently, there are no specific formulas for HF and the selection will be based on the type of malnutrition, the patient’s clinical situation, and associated comorbidities. Although there is some evidence pointing to the potential benefits of some nutrients, such as omega-3 fatty acids or coenzyme Q10, more studies are needed to be able to give a clear recommendation in this regard.

We should not forget the important role of cardiac rehabilitation in the approach to HF, especially in patients with sarcopenia, due to its capacity to improve symptomatology, increase exercise tolerance, or reduce hospitalizations due to HF.

Finally, we must emphasize that there is still a lack of awareness among professionals and patients about the relevance of malnutrition in the evolution and prognosis of HF. Our proposal includes encouraging routine screening for malnutrition in these patients, with validated tools such as MNA-SF or SGA to provide more reliable data on its prevalence. We would also encourage research to shed light on the usefulness of the GLIM criteria or body composition techniques, to establish specific cut-off points in this population, and to determine the ideal composition of nutritional formulas in this pathology. It would also be essential to conduct randomized, well-controlled clinical trials to assess the impact of the nutritional intervention in malnourished FH patients, especially in the out-of-hospital setting, where there is much less information. All this evidence could culminate in the development of evidence-based clinical practice guidelines that would allow standardization in the approach to patients with malnutrition and HF.

## Figures and Tables

**Table 1:** Summary of nutritional requirements in patients with HF.

Nutritional Needs		Recommendations	Effects
Caloric needs		22 kcal/kg (per actual weight) in normally nourished patients 24 kcal/kg per actual weight in malnourished patients	An adequate intake has been shown to improve quality of life. A reduced or excessive intake can cause decompensated HF
Protein needs		1.1 to 1.4 g/kg/day using actual weight	It promotes muscle synthesis, improving physical capacity and muscle mass
Liquids		1.5 to 2 L per day * (taking all daily fluid intake into account)	Adjust according to acute or chronic phase or hyponatremia
Sodium		2–3 g/day	Adjust according to acute or chronic phase or hyponatremia
Other electrolytes (potassium, magnesium, calcium)		Only if there is a deficiency (with oral supplementation and/or increased intake of electrolyte-rich foods)	Frequent deficiency due to the use of diuretics. Severe hypocalcemia can cause cardiac dysfunction.
Fat-soluble vitamins (A,D,E,K) and water-soluble vitamins (B6,B12)		Supplement if deficiency of “general recommended daily doses”.	Less frequent deficiencies, except for vitamin D deficiency.
Iron		If there is a deficiency (ferritin <100 ng/mL or 100–300 ng/mL with TSI < 20%). Administer iv	Deficiency can worsen HF functional status and quality of life.
Thiamine		If there is a deficiency, correct according to general recommendations. Low levels can worsen heart function	Decreased heart function. Severe deficiency leads to reversible cardiomyopathy
Coenzyme Q10		Its use may be considered. Variable dose (60 to 300 mg/day)	Deficiency associated with poorer cardiac function and biomarkers
PUFA: Omega 3		It could be considered (dose 1 to 2 mg/day). It may reduce the risk of admission for HF and/or CV death	A deficiency can increase mortality and readmissions in HF
Other micronutrients	Zinc, selenium, folate	Only if there is a deficiency (with oral supplementation and/or increased intake of electrolyte-rich foods)	Deficiency related to increased renal excretion. Severe selenium deficiency leads to reversible cardiomyopathy

kcal: kilocalorie; kg: kilograms; * Except for restriction in acute decompensation; TSAT: transferrin saturation index, iv: intravenous; PUFA: polyunsaturated fatty acids; HF: heart failure, CV: cardiovascular.

**Table 2:** Global and multiparametric assessment of malnutrition.

1 Phenotypic Criterion + 1 Etiological Criterion = Diagnosis of Malnutrition					
	Phenotypic Criteria			Etiological Criteria	
	Weight loss (%)	Low BMI (kg/m^2^)	Reduced muscle mass	Reduced dietary intake or absorption	Inflammation
Moderate malnutrition	5–10% in the last 6 months or 10–20% in >6 months	<20 in <70 years or <22 in ≥70 years	Mild to moderate deficiency *	≤50% of energy requirements or any reduction over >2 weeks or any condition that affects food absorption	Related to an acute illness or injury Related to chronic disease
Severe malnutrition	>10% in the last 6 months or >20% in more than 6 months	<18.5 in <70 years or <20 in ≥70 years	Severe deficiency *		

Source: own data. * Based on validated body composition techniques or anthropometric measurements, such as arm or calf circumference and hand grip strength as a supporting method.

**Table 3:** Advantages and disadvantages of the different items for assessing nutritional status.

	Advantages	Disadvantages
Anthropometry	Economical Accessible	Subclinical volume overload and changes in hydration status due to worsening of HF or in response to therapy complicate assessment.
Bioimpedance	Fast Does not involve radiation Inexpensive Easy to use	Estimation of body composition based on mathematical calculations. Limited use in patients with abnormal blood volume Contraindicated in patients with implantable cardiac devices
DXA	Easy to use Lower cost than CT or MRI. It permits the assessment of the 3 compartments: muscle, adipose, and bone.	Radiation exposure (albeit minimal) Major changes in hydration status (>5%) may overestimate fat-free mass.
CT	It permits the analysis of muscle mass, fat mass, and the distribution thereof (subcutaneous, visceral, and intramuscular)	High cost Radiation exposure
Muscle ultrasound	Simple Low cost Good correlation with the data obtained by MRI.	Cut-off points for low muscle mass are not universally defined.

HF: heart failure; MRI: magnetic resonance imaging; CT: computerized tomography.

**Table 4:** Nutritional recommendations to optimize intake in patients with HF.

***Weight Loss and Early Satiety***
Low-volume meals divided into 5–6 meals
Low-volume and calorie- and protein-enriched dishes
Liberalized diet according to patient preferences
Drink fluids between meals
Foods that are easy to chew and swallow
***Difficulty Chewing***
Cook food thoroughly and avoid tougher foods
Foods that are easy to chew and swallow
***Difficulty Swallowing***
Homogeneous diet with cream texture
Liquids with thickeners
***Nausea or Dyspepsia***
Easily-digestible diet
Eliminate foods that cause symptoms
***Altered Intestinal Transit***
Increase dietary fiber

**Table 5:** Oral enteral nutrition in patients with heart failure.

			Protein Malnutrition	Protein-Energy Malnutrition	Malnutrition with Altered Bowel Habits	Protein Malnutrition with Obesity
General			Complete diet Normocaloric Hyperproteic With fiber	Complete diet Hypercaloric Hyperproteic With fiber	Complete diet Normocaloric Hyperproteic With fiber, without fiber, or with soluble fiber	Protein module
Specific	Metabolic Disorders - Diabetes - Insulin resistance		Complete diet Normocaloric Hyperproteic With fiber	Complete diet Hypercaloric Hyperproteic With fiber	Complete diet Normocaloric Hyperproteic Without fiber, or with soluble fiber	Protein module
	Patient with Kidney Disease	Pre-dialysis	Complete diet Hypercaloric Hypoproteic With fiber	Complete diet Hypercaloric Hypoproteic With fiber	Complete diet Normocaloric Hypoproteic Without fiber	
		Dialysis	Complete diet Normocaloric Hyperproteic With fiber	Complete diet Hypercaloric Hyperproteic With fiber	Complete diet Normocaloric Hyperproteic Without fiber	Protein module
Dysphagia			Complete diet Modified-texture Hypercaloric and Hyperproteic	Complete diet Modified-texture Hypercaloric and Hyperproteic	Complete diet Modified-texture Hypercaloric and Hyperproteic	Diluted protein module and thickened to modified texture

**Table 7:** Rehabilitation indications and contraindications in HF.

Screening Criteria
Patients with HF regardless of LVEF, stable, NYHA functional class I–IV, with optimal medical treatment (including stable patients for whom treatment optimization is being completed), without contraindications or limitations for physical exercise.
Exclusion Criteria
Contraindication for physical exercise: Severe left ventricular outflow tract obstruction: severe aortic stenosis, severe hypertrophic obstructive cardiomyopathy. Advanced atrioventricular block.
Temporary contraindications.
Uncontrolled diabetes mellitus. Uncontrolled arterial hypertension. Uncontrolled arrhythmias. Myocarditis or pericarditis. Systemic infection. If <48 h have elapsed since an acute coronary syndrome. Intracardiac thrombus
Other
Partial or total dependency with scant family support or any physical, mental, or social disability preventing them from committing to carrying out the program.

LVEF: left ventricular ejection fraction; NYHA: New York Heart Association.

## Supplementary Materials

The following supporting information can be downloaded at: https://www.mdpi.com/article/10.3390/jcm12093320/s1, Table S1: General dietary recommendations to improve cardiovascular health. Table S2: Differential characteristics between the DASH diet and the Mediterranean diet.

## Abbreviations

CKD	chronic kidney disease
CV	cardiovascular
HF	heart failure
LVEF	left ventricular ejection fraction
MNA	Mini Nutritional Assessment
RAAS	renin-angiotensin-aldosterone system

## References

[1] McDonagh T.A., Metra M., Adamo M., Gardner R.S., Baumbach A., Böhm M., Burri H., Butler J., Čelutkienė J., Chioncel O. (2021). 2021 ESC Guidelines for the diagnosis and treatment of acute and chronic Heart failure. Eur. Heart J..

[2] Vest A.R., Chan M., Deswal A., Givertz M.M., Lekavich C., Lennie T., Litwin S.E., Parsly L., Rodgers J.E., Rich M.W. (2019). Nutrition, Obesity, and Cachexia in Patients With Heart Failure: A Consensus Statement from the Heart Failure Society of America Scientific Statements Committee. J. Card Fail.

[3] Eckel R.H., Jakicic J.M., Ard J.D., de Jesus J.M., Miller N.H., Hubbard V.S., Lee I.-M., Lichtenstein A.H., Loria C.M., Millen B.E. (2014). 2013 AHA/ACC guideline on lifestyle management to reduce cardiovascular risk: A report of the American College of Cardiology/American Heart Association Task Force on Practice Guidelines. Circulation.

[4] Cereda E., Pedrolli C., Klersy C., Bonardi C., Quarleri L., Cappello S., Turri A., Rondanelli M., Caccialanza R. (2016). Nutritional status in older persons according to healthcare setting: A systematic review and meta-analysis of prevalence data using MNA^®^. Clin. Nutr..

[5] Volkert D., Beck A.M., Cederholm T., Cruz-Jentoft A., Hooper L., Kiesswetter E., Maggio M., Raynaud-Simon A., Sieber C., Sobotka L. (2022). ESPEN practical guideline: Clinical nutrition and hydration in geriatrics. Clin. Nutr..

[6] Kaiser M.J., Bauer J.M., Rämsch C., Uter W., Guigoz Y., Cederholm T., Thomas D.R., Anthony P.S., Charlton K.E., Maggio M. (2010). Frequency of malnutrition in older adults: A multinational perspective using the mini nutritional assessment. J. Am. Geriatr. Soc..

[7] Sze S., Pellicori P., Zhang J., Clark A.L. (2019). Malnutrition, congestion and mortality in ambulatory patients with Heart failure. Heart.

[8] Wawrzeńczyk A., Anaszewicz M., Wawrzeńczyk A., Budzyński J. (2019). Clinical significance of nutritional status in patients with chronic Heart failure-a systematic review. Heart Fail Rev..

[9] von Haehling S., Lainscak M., Springer J., Anker S.D. (2009). Cardiac cachexia: A systematic overview. Pharmacol. Ther..

[10] Sager R., Güsewell S., Rühli F., Bender N., Staub K. (2002). Obesity and the risk of Heart failure. N. Engl. J. Med..

[11] Sánchez M.A., Rodríguez J.L.L., Freire R.B., Colet J.C., Leiro M.G.C., Vílchez F.G., Lorite N.M., Cubero J.S., Mateas F.R., Somoza F.J.E. (2016). Classification and Quality Standards of Heart Failure Units: Scientific Consensus of the Spanish Society of Cardiology. Rev. Esp. Cardiol..

[12] Cederholm T., Barazzoni R., Austin P., Ballmer P., Biolo G., Bischoff S.C., Compher C., Correia I., Higashiguchi T., Holst M. (2017). ESPEN guidelines on definitions and terminology of clinical nutrition. Clin. Nutr..

[13] Muscaritoli M., Anker S.D., Argilés J., Aversa Z., Bauer J.M., Biolo G., Boirie Y., Bosaeus I., Cederholm T., Costelli P. (2010). Consensus definition of sarcopenia, cachexia and pre-cachexia: Joint document elaborated by Special Interest Groups (SIG) “cachexia-anorexia in chronic wasting diseases” and “nutrition in geriatrics”. Clin. Nutr..

[14] Anker S.D., Ponikowski P., Varney S., Chua T.P., Clark A.L., Webb-Peploe K.M., Harrington D., Kox W.J., Poole-Wilson P.A., Coats A.J. (1997). Wasting as independent risk factor for mortality in chronic Heart failure. Lancet.

[15] Cruz-Jentoft A.J., Bahat G., Bauer J., Boirie Y., Bruyère O., Cederholm T., Cooper C., Landi F., Rolland Y., Sayer A.A. (2019). Sarcopenia: Revised European consensus on definition and diagnosis. Age. Ageing.

[16] Emami A., Saitoh M., Valentova M., Sandek A., Evertz R., Ebner N., Loncar G., Springer J., Doehner W., Lainscak M. (2018). Comparison of sarcopenia and cachexia in men with chronic Heart failure: Results from the Studies Investigating Co-morbidities Aggravating Heart Failure (SICA-HF). Eur. J. Heart Fail.

[17] Kirkman D.L., Bohmke N., Billingsley H.E., Carbone S. (2020). Sarcopenic Obesity in Heart Failure With Preserved Ejection Fraction. Front. Endocrinol..

[18] Bonilla-Palomas J.L., Gámez-López A.L., Anguita-Sánchez M.P., Castillo-Domínguez J.C., García-Fuertes D., Crespin-Crespin M., López-Granados A., de Lezo J.S. (2011). Impact of malnutrition on long-term mortality in hospitalized patients with Heart failure. Rev. Esp. Cardiol..

[19] Sze S., Pellicori P., Kazmi S., Rigby A., Cleland J.G., Wong K., Clark A.L. (2018). Prevalence and Prognostic Significance of Malnutrition Using 3 Scoring Systems Among Outpatients With Heart Failure: A Comparison With Body Mass Index. JACC Heart Fail.

[20] Suzuki T., Palus S., Springer J. (2018). Skeletal muscle wasting in chronic Heart failure. ESC Heart Fail.

[21] Packer M. (1992). The neurohormonal hypothesis: A theory to explain the mechanism of disease progression in Heart failure. J. Am. Coll. Cardiol..

[22] Josiak K., Jankowska E.A., Piepoli M.F., Banasiak W., Ponikowski P. (2014). Skeletal myopathy in patients with chronic Heart failure: Significance of anabolic-androgenic hormones. J. Cachexia Sarcopenia Muscle.

[23] Rahman A., Jafry S., Jeejeebhoy K., Nagpal A.D., Pisani B., Agarwala R. (2016). Malnutrition and Cachexia in Heart Failure. JPEN J. Parenter. Enteral. Nutr..

[24] Battin D.L., Ali S., Shahbaz A.U., Munir A., Davis R.C., Newman K.P., Weber K.T., Massie J.D. (2010). Hypoalbuminemia and lymphocytopenia in patients with decompensated biventricular failure. Am. J. Med. Sci..

[25] Hesse B., Parving H.H., Lund Jacobsen H., Noer I. (1976). Transcapillary escape rate of albumin and right atrial pressure in chronic congestive heart failure before and after treatment. Circ. Res..

[26] Wrigley B.J., Lip G.Y.H., Shantsila E. (2011). The role of monocytes and inflammation in the pathophysiology of heart failure. Eur. J. Heart Fail.

[27] Floras J.S. (2009). Sympathetic nervous system activation in human heart failure: Clinical implications of an updated model. J. Am. Coll. Cardiol..

[28] Schrier R.W., Abraham W.T. (1999). Hormones and hemodynamics in heart failure. N. Engl. J. Med..

[29] Hryniewicz K., Androne A.S., Hudaihed A., Katz S.D. (2003). Partial reversal of cachexia by beta-adrenergic receptor blocker therapy in patients with chronic Heart failure. J. Card Fail.

[30] Anker S.D., Chua T.P., Ponikowski P., Harrington D., Swan J.W., Kox W.J., Poole-Wilson P.A., Coats A.S. (1997). Hormonal changes and catabolic/anabolic imbalance in chronic Heart failure and their importance for cardiac cachexia. Circulation.

[31] Pocock S.J., McMurray J.J., Dobson J., Yusuf S., Granger C.B., Michelson E.L., Östergren J., Pfeffer M.A., Solomon S.D., Anker S.D. (2008). Weight loss and mortality risk in patients with chronic Heart failure in the candesartan in Heart failure: Assessment of reduction in mortality and morbidity (CHARM) programme. Eur. Heart J..

[32] Berry C., Clark A.L. (2000). Catabolism in chronic Heart failure. Eur. Heart J..

[33] Ballmer P.E. (2001). Causes and mechanisms of hypoalbuminaemia. Clin. Nutr..

[34] Von Haehling S., Ebner N., Dos Santos M.R., Springer J., Anker S.D. (2017). Muscle wasting and cachexia in Heart failure: Mechanisms and therapies. Nat. Rev. Cardiol..

[35] Lund L.H., Williams J.J., Freda P., Lamanca J.J., Lejemtel T.H., Mancini D.M. (2009). Ghrelin resistance occurs in severe *Heart* failure and resolves after Heart transplantation. Eur. J. Heart Fail.

[36] Breitbart A., Auger-Messier M., Molkentin J.D., Heineke J. (2011). Myostatin from the Heart: Local and systemic actions in cardiac failure and muscle wasting. Am. J. Physiol. Heart Circ. Physiol..

[37] Sharma A., Stevens S.R., Lucas J., Fiuzat M., Adams K.F., Whellan D.J., Donahue M.P., Kitzman D.W., Piña I.L., Zannad F. (2017). Utility of Growth Differentiation Factor-15, A Marker of Oxidative Stress and Inflammation, in Chronic Heart Failure: Insights From the HF-ACTION Study. JACC Heart Fail.

[38] Heidenreich P.A., Bozkurt B., Aguilar D., Allen L.A., Byun J.J., Colvin M.M., Deswal A., Drazner M.H., Dunlay S.M., Evers L.R. (2022). 2022 AHA/ACC/HFSA Guideline for the Management of Heart Failure: A Report of the American College of Cardiology/American Heart Association Joint Committee on Clinical Practice Guidelines. Circulation.

[39] Sciatti E., Lombardi C., Ravera A., Vizzardi E., Bonadei I., Carubelli V., Gorga E., Metra M. (2016). Nutritional deficiency in patients with Heart failure. Nutrients.

[40] Mijan de la Torre A., de Mateo Silleras B., AM P.G. (2017). Nutrición e insuficiencia cardiaca. Nutricion e Insuficiencia Cardiaca En: Ángel Gil Tratado de Nutrición Tomo 5: Nutrición y Enfermedad.

[41] Kuehneman T., Gregory M., de Waal D., Davidson P., Frickel R., King C., Gradwell E., Handu D. (2018). Academy of Nutrition and Dietetics Evidence-Based Practice Guideline for the Management of Heart Failure in Adults. J. Acad. Nutr. Diet..

[42] Olveira G., González Romero S. (2010). Nutrición en el adulto. Tratado de Nutrición En: Gil Hernández A (Dir).

[43] A Ezekowitz J., Colin-Ramirez E., Ross H., Escobedo J., Macdonald P., Troughton R., Saldarriaga C., Alemayehu W., A McAlister F., Arcand J. (2022). Reduction of dietary sodium to less than 100 mmol in Heart failure (SODIUM-HF): An international, open-label, randomised, controlled trial. Lancet.

[44] Allard M.L., Jeejeebhoy K.N., Sole M.J. (2006). The management of conditioned nutritional requirements in Heart failure. Heart Fail Rev..

[45] Lennie T.A., Andreae C., Rayens M.K., Song E.K., Dunbar S.B., Pressler S.J., Heo S., Kim J., Moser D.K. (2018). Micronutrient Deficiency Independently Predicts Time to Event in Patients With Heart Failure. J. Am. Heart Assoc..

[46] Fernández-Pombo A., Rodríguez-Carnero G., Castro A.I., Cantón-Blanco A., Seoane L.M., Casanueva F.F., Crujeiras A.B., Martínez-Olmos M.A. (2021). Relevance of nutritional assessment and treatment to counteract cardiac cachexia and sarcopenia in chronic Heart failure. Clin. Nutr..

[47] McKeag N.A., McKinley M.C., Harbinson M.T., Noad R.L., Dixon L.H., McGinty A., Neville C.E., Woodside J.V., McKeown P.P. (2014). The effect of multiple micronutrient supplementation on left ventricular ejection fraction in patients with chronic stable Heart failure: A randomized, placebo-controlled trial. JACC Heart Fail.

[48] Kassis N., Hariri E.H., Karrthik A.K., Ahuja K.R., Layoun H., Saad A.M., Gad M.M., Kaur M., Bazarbashi N., Griffin B.P. (2022). Supplemental calcium and vitamin D and long-term mortality in aortic stenosis. Heart.

[49] Jain A., Mehta R., Al-Ani M., Hill J.A., Winchester D.E. (2015). Determining the Role of Thiamine Deficiency in Systolic Heart Failure: A Meta-Analysis and Systematic Review. J. Card Fail.

[50] Tavazzi L., Maggioni A.P., Marchioli R., Barlera S., Franzosi M.G., Latini R., Lucci D., Nicolosi G.L., Porcu M., Tognoni G. (2008). Effect of n-3 polyunsaturated fatty acids in patients with chronic Heart failure (the GISSI-HF trial): A randomised, double-blind, placebo-controlled trial. Lancet.

[51] Nodari S., Triggiani M., Campia U., Manerba A., Milesi G., Cesana B.M., Gheorghiade M., Cas L.D. (2011). Effects of n-3 polyunsaturated fatty acids on left ventricular function and functional capacity in patients with dilated cardiomyopathy. J. Am. Coll. Cardiol..

[52] Kojuri J., Ostovan M., Rezaian G.R., Dialameh P.A., Zamiri N., Sharifkazemi M., Jannati M. (2013). Effect of omega-3 on brain natriuretic peptide and echocardiographic findings in Heart failure: Double-blind placebo-controlled randomized trial. J. Cardiovasc. Dis. Res..

[53] Masson S., Marchioli R., Mozaffarian D., Bernasconi R., Milani V., Dragani L., Tacconi M., Marfisi R.M., Borgese L., Cirrincione V. (2013). Plasma n-3 polyunsaturated fatty acids in chronic Heart failure in the GISSI-Heart Failure Trial: Relation with fish intake, circulating biomarkers, and mortality. Am. Heart J..

[54] Keogh A., Fenton S., Leslie C., Aboyoun C., Macdonald P., Zhao Y.C., Bailey M., Rosenfeldt F. (2003). Randomised double-blind, placebo-controlled trial of coenzyme, Q.; therapy in class II and III systolic *Heart* failure. Heart Lung. Circ..

[55] Mortensen S.A., Rosenfeldt F., Kumar A., Dolliner P., Filipiak K.J., Pella D., Alehagen U., Steurer G., Littarru G.P. (2014). The effect of coenzyme Q10 on morbidity and mortality in chronic Heart failure: Results from Q-SYMBIO: A randomized double-blind trial. JACC Heart Fail.

[56] Fotino A.D., Thompson-Paul A.M., Bazzano L.A. (2013). Effect of coenzyme Q_10_ supplementation on Heart failure: A meta-analysis. Am. J. Clin. Nutr..

[57] Lin H., Zhang H., Lin Z., Li X., Kong X., Sun G. (2016). Review of nutritional screening and assessment tools and clinical outcomes in Heart failure. Heart Fail Rev..

[58] Lv S., Ru S. (2021). The prevalence of malnutrition and its effects on the all-cause mortality among patients with Heart failure: A systematic review and meta-analysis. PLoS ONE.

[59] Imoberdorf R., Meier R., Krebs P., Hangartner P.J., Hess B., Stäubli M., Wegmann D., Rühlin M., Ballmer P.E. (2010). Prevalence of undernutrition on admission to Swiss hospitals. Clin. Nutr..

[60] Norman K., Pichard C., Lochs H., Pirlich M. (2008). Prognostic impact of disease-related malnutrition. Clin. Nutr..

[61] Visseren F.L.J., Mach F., Smulders Y.M., Carballo D., Koskinas K.C., Bäck M., Benetos A., Biffi A., Boavida J.-M., Capodanno D. (2021). 2021 ESC Guidelines on cardiovascular disease prevention in clinical practice. Eur. Heart J..

[62] House A.A., Wanner C., Sarnak M.J., Piña I.L., McIntyre C.W., Komenda P., Kasiske B.L., Deswal A., Defilippi C.R., Cleland J.G.F. (2019). Heart failure in chronic kidney disease: Conclusions from a Kidney Disease: Improving Global Outcomes (KDIGO) Controversies Conference. Kidney Int..

[63] Fouque D., Kalantar-Zadeh K., Kopple J., Cano N., Chauveau P., Cuppari L., Franch H., Guarnieri G., Ikizler T.A., Kaysen G. (2008). A proposed nomenclature and diagnostic criteria for protein-energy wasting in acute and chronic kidney disease. Kidney Int..

[64] Ramos C.I., González-Ortiz A., Espinosa-Cuevas A., Avesani C.M., Carrero J.J., Cuppari L. (2021). Does dietary potassium intake associate with hyperkalemia in patients with chronic kidney disease?. Nephrol. Dial. Transplant.

[65] Moustafa F., Dopeux L., Mulliez A., Boirie Y., Morand C., Gentes E., Farigon N., Richard D., Lebreton A., Teissandier D. (2021). Severe undernutrition increases bleeding risk on vitamin-K antagonists. Clin. Nutr..

[66] Kawai M., Harada M., Motoike Y., Koshikawa M., Ichikawa T., Watanabe E., Ozaki Y. (2019). Impact of serum albumin levels on supratherapeutic PT-INR control and bleeding risk in atrial fibrillation patients on warfarin: A prospective cohort study. Int. J. Cardiol. Heart Vasc..

[67] Violi F., Lip G.Y.H., Pignatelli P., Pastori D. (2016). Interaction Between Dietary Vitamin K Intake and Anticoagulation by Vitamin K Antagonists: Is It Really True?: A Systematic Review. Medicine.

[68] Chen A., Stecker E., Warden B.A. (2020). Direct Oral Anticoagulant Use: A Practical Guide to Common Clinical Challenges. J. Am Heart Assoc..

[69] Joaquín C., Puig R., Gastelurrutia P., Lupón J., de Antonio M., Domingo M., Moliner P., Zamora E., Martin M., Alonso N. (2019). Mini nutritional assessment is a better predictor of mortality than subjective global assessment in Heart failure out-patients. Clin. Nutr..

[70] Cederholm T., Jensen G.L., Correia M.I.T.D., Gonzalez M.C., Fukushima R., Higashiguchi T., Baptista G., Barazzoni R., Blaauw R., Coats A.J. (2019). GLIM criteria for the diagnosis of malnutrition—A consensus report from the global clinical nutrition community. Clin. Nutr..

[71] Hirose S., Matsue Y., Kamiya K., Kagiyama N., Hiki M., Dotare T., Sunayama T., Konishi M., Saito H., Saito K. (2021). Prevalence and prognostic implications of malnutrition as defined by GLIM criteria in elderly patients with Heart failure. Clin. Nutr..

[72] Joaquín C., Alonso N., Lupón J., Gastelurrutia P., Pérez-Monstesdeoca A., Domingo M., Zamora E., Socias G., Ramos A., Bayes-Genis A. (2022). Nutritional Status According to the GLIM Criteria in Patients with Chronic Heart Failure: Association with Prognosis. Nutrients.

[73] Castillo-Martinez L., Colin-Ramirez E., Orea-Tejeda A., Islas D.G.G., Rodríguez-García W., Díaz C.S., Rodríguez A.E.G., Durán M.V., Davies C.K. (2012). Cachexia assessed by bioimpedance vector analysis as a prognostic indicator in chronic stable Heart failure patients. Nutrition.

[74] Prado C.M.M., Heymsfield S.B. (2014). Lean tissue imaging: A new era for nutritional assessment and intervention. JPEN J. Parenter. Enteral. Nutr..

[75] Fuentes-Abolafio I.J., Ricci M., Bernal-López M.R., Gómez-Huelgas R., Cuesta-Vargas A.I., Pérez-Belmonte L.M. (2022). Biomarkers and the quadriceps femoris muscle architecture assessed by ultrasound in older adults with Heart failure with preserved ejection fraction: A cross-sectional study. Aging Clin. Exp. Res..

[76] Chung C.J., Wu C., Jones M., Kato T.S., Dam T.T., Givens R.C., Templeton D.L., Maurer M.S., Naka Y., Takayama H. (2014). Reduced handgrip strength as a marker of frailty predicts clinical outcomes in patients with Heart failure undergoing ventricular assist device placement. J. Card Fail.

[77] Tran R.H., Aldemerdash A., Chang P., Sueta C.A., Kaufman B., Asafu-Adjei J., Vardeny O., Daubert E., Alburikan K.A., Kucharska-Newton A.M. (2018). Guideline-Directed Medical Therapy and Survival Following Hospitalization in Patients with Heart Failure. Pharmacotherapy.

[78] Papadaki A., Martínez-González M., Alonso-Gómez  A., Rekondo  J., Salas-Salvadó  J., Corella  D., Ros  E., Fitó  M., Estruch  R., Lapetra  J. (2017). Mediterranean diet and risk of Heart failure: Results from the PREDIMED randomized controlled trial. Eur. J. Heart Fail.

[79] Miró Ò., Estruch R., Martín-Sánchez F.J., Gil V., Jacob J., Herrero-Puente P., Mateo S.H., Aguirre A., Andueza J.A., Llorens P. (2018). Adherence to Mediterranean Diet and All-Cause Mortality After an Episode of Acute Heart Failure: Results of the MEDIT-AHF Study. JACC Heart Fail.

[80] Lara K.M., Levitan E.B., Gutierrez O.M., Shikany J.M., Safford M.M., Judd S.E., Rosenson R.S. (2019). Dietary Patterns and Incident Heart Failure in US Adults Without Known Coronary Disease. J. Am. Coll. Cardiol..

[81] Levitan E.B., Wolk A., Mittleman M.A. (2009). Consistency with the DASH diet and incidence of Heart failure. Arch. Intern. Med..

[82] Hummel S.L., Karmally W., Gillespie B.W., Helmke S., Teruya S., Wells J., Trumble E., Jimenez O., Marolt C., Wessler J.D. (2018). Home-Delivered Meals Postdischarge From Heart Failure Hospitalization. Circ. Heart Fail.

[83] Tektonidis T.G., Åkesson A., Gigante B., Wolk A., Larsson S.C. (2016). Adherence to a Mediterranean diet is associated with reduced risk of Heart failure in men. Eur. J. Heart Fail.

[84] Tektonidis T.G., Åkesson A., Gigante B., Wolk A., Larsson S.C. (2015). A Mediterranean diet and risk of myocardial infarction, Heart failure and stroke: A population-based cohort study. Atherosclerosis.

[85] Horwich T.B., Fonarow G.C., Hamilton M.A., MacLellan W.R., Woo M.A., Tillisch J.H. (2001). The relationship between obesity and mortality in patients with Heart failure. J. Am. Coll. Cardiol..

[86] Frías L., Cuerda C. (2014). Nutrición enteral; indicaciones, sondas y materiales. Nutr. Hosp..

[87] Compher C., Bingham A.L., McCall M., Patel J., Rice T.W., Braunschweig C., McKeever L. (2022). Guidelines for the provision of nutrition support therapy in the adult critically ill patient: The American Society for Parenteral and Enteral Nutrition. JPEN J. Parenter. Enteral. Nutr..

[88] Weimann A., Braga M., Carli F., Higashiguchi T., Hübner M., Klek S., Laviano A., Ljungqvist O., Lobo D.N., Martindale R.G. (2021). ESPEN practical guideline: Clinical nutrition in surgery. Clin. Nutr..

[89] Jiménez Jiménez F.J., Cervera Montes M., Blesa Malpica A.L. (2011). Guidelines for specialized nutritional and metabolic support in the critically-ill patient. Update. Consensus of the Spanish Society of Intensive Care Medicine and Coronary Units-Spanish Society of Parenteral and Enteral Nutrition (SEMICYUC-SENPE): Card. Med. Intensiva..

[90] Bonilla-Palomas J.L., Gámez-López A.L., Castillo-Domínguez J.C., Moreno-Conde M., Ibáñez M.C.L., Expósito R.A., Ortega E.R., Anguita-Sánchez M.P., Villar-Ráez A. (2016). Nutritional Intervention in Malnourished Hospitalized Patients with Heart Failure. Arch. Med. Res..

[91] Habaybeh D., de Moraes M.B., Slee A., Avgerinou C. (2021). Nutritional interventions for Heart failure patients who are malnourished or at risk of malnutrition or cachexia: A systematic review and meta-analysis. Heart Fail Rev..

[92] Hersberger L., Dietz A., Bürgler H., Bargetzi A., Bargetzi L., Kägi-Braun N., Tribolet P., Gomes F., Hoess C., Pavlicek V. (2021). Individualized Nutritional Support for Hospitalized Patients With Chronic Heart Failure. J. Am. Coll. Cardiol..

[93] Billingsley H.E., Hummel S.L., Carbone S. (2020). The role of diet and nutrition in Heart failure: A state-of-the-art narrative review. Prog. Cardiovasc. Dis..

[94] Driggin E., Cohen L.P., Gallagher D., Karmally W., Maddox T., Hummel S.L., Carbone S., Maurer M.S. (2022). Nutrition Assessment and Dietary Interventions in Heart Failure: JACC Review Topic of the Week. J. Am. Coll. Cardiol..

[95] Schuetz P., Sulo S., Walzer S., Krenberger S., Stagna Z., Gomes F., Mueller B., Brunton C. (2022). Economic Evaluation of Individualized Nutritional Support for Hospitalized Patients with Chronic Heart Failure. Nutrients.

[96] Little M.O. (2018). Updates in nutrition and polypharmacy. Curr. Opin Clin. Nutr. Metab. Care..

[97] Zenuk C., Healey J., Donnelly J., Vaillancourt R., Almalki Y., Smith S. (2003). Thiamine deficiency in congestive Heart failure patients receiving long term furosemide therapy. Can. J. Clin. Pharmacol..

[98] Ahmed A., Zannad F., E Love T., Tallaj J., Gheorghiade M., Ekundayo O.J., Pitt B. (2007). A propensity-matched study of the association of low serum potassium levels and mortality in chronic Heart failure. Eur. Heart J..

[99] Esteban-Fernández A., Salinas G.A., de Juan Bagudá J., Fernández-Fresnedo G., Magaña J.G., Iniesta Á.M., Rivera-Juárez A., Marcos M.C. (2021). Fisiopatología, diagnóstico y tratamiento de la hipomagnesemia en pacientes con insuficiencia cardiaca. REC CardioClinics.

[100] McColl K.E.L. (2009). Effect of proton pump inhibitors on vitamins and iron. Am. J. Gastroenterol..

[101] Fenton R., Brook-Barclay L., Delaney C.L., Spark J.I., Miller M.D. (2016). Do Medications Commonly Prescribed to Patients with Peripheral Arterial Disease Have an Effect on Nutritional Status? A Review of the Literature. Ann. Vasc. Surg..

[102] Edelmann F., Gelbrich G., Düngen H.-D., Fröhling S., Wachter R., Stahrenberg R., Binder L., Töpper A., Lashki D.J., Schwarz S. (2011). Exercise training improves exercise capacity and diastolic function in patients with Heart failure with preserved ejection fraction: Results of the Ex-DHF (Exercise training in Diastolic Heart Failure) pilot study. J. Am. Coll. Cardiol..

[103] Ismail H., McFarlane J.R., Nojoumian A.H., Dieberg G., Smart N.A. (2013). Clinical outcomes and cardiovascular responses to different exercise training intensities in patients with Heart failure: A systematic review and meta-analysis. JACC Heart Fail.

[104] Taylor R.S., Walker S., Smart N.A., Piepoli M.F., Warren F.C., Ciani O., O’Connor C., Whellan D., Keteyian S.J., Coats A. (2018). Impact of exercise-based cardiac rehabilitation in patients with Heart failure (ExTraMATCH II) on mortality and hospitalisation: An individual patient data meta-analysis of randomised trials. Eur. J. Heart Fail.

[105] Taylor R.S., Walker S., Smart N.A., Piepoli M.F., Warren F.C., Ciani O., Whellan D., O’Connor C., Keteyian S.J., Coats A. (2019). Impact of Exercise Rehabilitation on Exercise Capacity and Quality-of-Life in Heart Failure: Individual Participant Meta-Analysis. J. Am. Coll. Cardiol..

[106] Valle A., Arrarte V., Pinilla J.M.G., Campuzano R., de Pablo C., Beltrán P., Garcia-Quintana A., Almenar L., Bover R., Ortiz C. (2020). Consenso de expertos en la asistencia multidisciplinaria y el abordaje integral de la insuficiencia cardiaca. Desde el alta hospitalaria hasta la continuidad asistencial con primaria. Rev. Española. Cardiol..

[107] Piepoli M.F., Conraads V., Corrà U., Dickstein K., Francis D.P., Jaarsma T., Mcmurray J., Pieske B., Piotrowicz E., Schmid J.-P. (2011). Exercise training in Heart failure: From theory to practice. A consensus document of the Heart Failure Association and the European Association for Cardiovascular Prevention and Rehabilitation. Eur. J. Heart Fail.

[108] Bozkurt B., Fonarow G.C., Goldberg L.R., Guglin M., Josephson R.A., Forman D.E., Lin G., Lindenfeld J., O’Connor C., Panjrath G. (2021). Cardiac Rehabilitation for Patients With Heart Failure: JACC Expert Panel. J. Am. Coll. Cardiol..

