# Supplementary material for: Diagnosis and Management of Malnutrition in Patients with Heart Failure
Source: J Clin Med. 2023 May 6;12(9):3320. doi: 10.3390/jcm12093320 (PMC10179706; doi:10.3390/jcm12093320)
Supplement: Supplementary file 1 [file jcm-12-03320-s001.zip › jcm-2372700-supplementary.pdf]

Supplementary material. Table S1. General dietary recommendations to improve cardiovascular health.

1. Adjust energy intake and expenditure to achieve and maintain an adequate body weight.
2. Eat abundant and varied fruit and vegetables.
3. Prioritize whole foods and products.
4. Choose healthy sources of protein<br>a. mainly vegetable proteins (legumes and nuts)<br>b. fish and shellfish<br>c. low-fat or fat-free dairy products instead of full-fat dairy products<br>d. if you eat meat or poultry, choose lean cuts and avoid processed food.
5. Use liquid vegetable oils instead of tropical oils (coconut, palm) and partially hydrogenated (trans) fats.
6. Choose minimally-processed foods instead of ultra-processed foods *.
7. Minimize the intake of drinks and foods with added sugars.
8. Choose and prepare foods with a reduced salt intake.
9. If you do not drink alcohol, do not start; if you choose to drink alcohol, limit your intake as much as possible.
10. Stick to these recommendations, regardless of where the food is prepared or eaten.

Adapted from Heidenreich PA and Vest AR [2,38].

*There is no commonly accepted definition for ultra-processed foods, and there may be healthy foods in the category of ultra-processed foods.

Supplementary material. Table S2. Differential characteristics between the DASH diet and the Mediterranean diet.

Parameters	Mediterranean diet	DASH diet
**Commonalities**	Basic consumption: whole grains, legumes, fruit, vegetables and nuts. Limited consumption: red meat, sweets, sugary drinks.	Basic consumption: whole grains, legumes, fruit and vegetables, seeds and nuts. Limited consumption: red meat, sweets, sugary drinks.
**Animal protein consumption**	It emphasizes the consumption of fish (especially oily) and lean meat.<br>It limits red and processed meat.	The consumption of poultry, lean meats and fish is recommended (without specifying white or oily).
**Alcohol consumption**	It recommends a moderate consumption of alcohol (wine)<br>There are new scores that do not consider moderate consumption as "healthy" and do not count it for the score.	It recommends reducing the consumption of alcohol.
**High-fat foods**	It does not restrict fat intake, but recommends a healthy lipid profile (more MUFA and PUFA: in the form of olive oil, oily fish, nuts, etc.)<br>Olive oil: phenolic compounds (hydroxytyrosol) help to prevent oxidative and anti-inflammatory damage.	It recommends a reduction in total fats, with the emphasis on saturated fats. It recommends: skimmed dairy products, reduction in red meat, etc.
**Salt intake**	None of the Mediterranean diet adherence scores address salt content, limiting only the consumption of red meat	It reduces the addition of salt (spices and other techniques are recommended for seasoning dishes) and limits high-salt

	and other high-salt products. Thus, consumption is reduced.	products. There are two restrictions: <2.3 g/d or <1.5 g/d (more restrictive)
**Score**	There are several methods of scoring diet adherence (PREDIMED and others), and there are discrepancies between the systems:<br>- Some systems rate the consumption of olive oil or foods high in MUFA (rather than the saturated fat ratio).<br>- Some do not include a score for alcohol consumption.<br>- Some include a score for a low consumption of sweets or for maintaining a moderate consumption of dairy products.	All the studies are performed with the same score.
